# Whole-Genome Re-Alignment Facilitates Development of Specific Molecular Markers for Races 1 and 4 of *Xanthomonas campestris* pv. *campestris*, the Cause of Black Rot Disease in *Brassica oleracea*

**DOI:** 10.3390/ijms18122523

**Published:** 2017-11-24

**Authors:** Mehede Hassan Rubel, Arif Hasan Khan Robin, Sathishkumar Natarajan, Joana G. Vicente, Hoy-Taek Kim, Jong-In Park, Ill-Sup Nou

**Affiliations:** 1 Department of Horticulture, Sunchon National University, 255, Jungang-ro, Suncheon 57922, Korea; mehede@nstu.edu.bd (M.H.R.); gpb21bau@gmail.com (A.H.K.R.); sathisbioinfo@gmail.com (S.N.); htkim@sunchon.ac.kr (H.-T.K.); 2 School of Life Sciences, University of Warwick, Wellesbourne Campus, Warwick CV35 9EF, UK; joana.vicente@warwick.ac.uk

**Keywords:** black rot, *Xanthomonas campestris* pv. *campestris*, races, PCR, SCAR, InDel, cabbage

## Abstract

Black rot, caused by *Xanthomonas campestris* pv. *campestris* (*Xcc*), is a seed borne disease of *Brassicaceae*. Eleven pathogenic races have been identified based on the phenotype interaction pattern of differential brassica cultivars inoculated with different strains. Race 1 and 4 are the two most frequent races found in *Brassica oleracea* crops. In this study, a PCR molecular diagnostic tool was developed for the identification of *Xcc* races 1 and 4 of this pathogen. Whole genomic sequences of races 1, 3, 4 and 9 and sequences of three other *Xanthomonas* pathovars/species (*X. campestris* pv. *incanae* (*Xci*), *X. campestris* pv. *raphani* (*Xcr*) and *X.* *euvesicatoria* (*Xev*) were aligned to identify variable regions among races. To develop specific markers for races 1 and 4, primers were developed from a region where sequences were dissimilar in other races. Sequence-characterized amplified regions (SCAR) and insertion or deletion of bases (InDel) were used to develop each specific set of primers. The specificity of the selected primers was confirmed by PCR tests using genomic DNA of seven different *Xcc* races, two strains of *X. campestris* pathovars and other species of bacteria. Bacterial samples of the races 1 and 4 isolates were collected from artificially inoculated cabbage leaves to conduct bio-PCR. Bio-PCR successfully detected the two *Xcc* isolates. By using our race-specific markers, a potential race 1 strain from the existing Korean *Xcc* collection was identified. The *Xcc* race 1 and 4-specific markers developed in this study are novel and can potentially be used for rapid detection of *Xcc* races through PCR.

## 1. Introduction

Black rot, a seed-borne disease caused by *Xanthomonas campestris* pv. *campestris* (*Xcc*), is one of the most important diseases of plants of the *Brassicaceae* family [1]. Black rot is a global problem which can reduce more than fifty percent yield under severe attacks in favorable conditions [2]. This pathogen is found all over the world, showing diversity in different countries and provinces of the same country [3,4,5,6].

*Xcc* is a small, rod-shaped, mobile, aerobic, gram-negative, non-spore forming obligate bacterium [7]. The pathogen enters the vascular tissues mainly through the hydathodes, but also through wounded tissues and stomata and is able to develop disease symptoms systemically. This disease is favored by warm, humid conditions and can spread rapidly by rain dispersal and irrigation water [8]. Infected seeds and plants, crop debris and cruciferous weeds are potential inoculum sources of this disease.

*Xcc* was grouped into six races based on a postulated gene-for-gene model [3,9]. Later, this model was expanded to include nine races [1,10]. Seven pathogenic races were identified on the basis of interaction between differential cultivars of *Brassica* species and different strains of *Xcc* [5]. Two novel races of this organism, race 10 and race 11, have been reported in Portugal this year [11]. Among these races, races 1 and 4 are the two most frequently found races all over the world largely because of their severe infections in *Brassica oleracea* crops [12].

Management of black rot is very difficult. The disease can be controlled by using healthy seeds, crop rotation and resistant cultivars [2,13], but in practice these methods have frequently not been efficiently used. In order to design effective control strategies, including seed health tests and development of resistant varieties against black rot disease, it is important to know the race of the pathogens present in different crops and areas. To date, a total of twenty-seven race-specific resistant genotypes have been developed in USA, Russia, UK, and Portugal (Table S1 [3,10,11,14,15]). No race-specific resistant cultivars of *Brassica oleracea* species have been developed for Korea as the disease has not been studied in this country yet. The identification of *Xcc* races and thereafter development of resistant cultivars of *Brassica oleracea* to black rot have become a priority for the vegetable breeders in Korea.

Developing race-specific markers is not only required to identify the races in a particular location, but also important for breeding programmes that aim to develop race-specific *B. oleracea* resistant genotypes for each area. Race-specific markers might also enable researchers to identify the presence of *Xcc* at an early stage. There are several methods used for the detection of *Xcc* such as selective and semi-selective media, plant bioassays [16,17] and serological techniques [18,19]. These techniques, however, were developed for *Xcc* detection and identification of *Xcc* at the pathovar and species level only. In addition, these methods are time consuming and labour intensive [20]. Recently, DNA-based techniques have been developed for *Xcc* detection. PCR is a very powerful, fast, reliable, and comparatively low cost method for the specific identification of plant pathogenic bacteria, yeast and also other organisms [21,22,23,24,25]. Sequence-characterized amplified region (SCAR) analysis can be a source of specific sequences for developing diagnostic molecular markers for detecting *Xcc* races 1 and 4 [26,27]. Other reliable molecular markers can be based on InDels, either insertions or deletions, that can be useful especially in phylogenetic studies in natural populations and also for species-identification procedures [28,29,30,31].

To date, a single method has yet to be developed for race-specific detection of *Xcc*. Therefore, it is important to develop race-specific markers that can rapid and efficiently identify *Xcc* races 1 and 4 from plant samples.

Until now, only pathogenicity tests on differential cultivars are being used for *Xcc* race-specific detection across the world (Table S2 [3,5,9,10,11,32]). PCR with race-specific markers could be used for faster determination of races. The objective of this work was to develop race 1 and 4 specific markers for identifying these races of *Xcc*. To achieve this goal, available *Xcc* and *Xanthomonas* species sequences were retrieved and aligned and race 1 and 4 specific primers were developed. The developed markers were validated for specificity to race 1 and 4 including bacterial DNA pools of *Xcc* races, *X. campestris* pathovars, other bacterial species and a protist.

## 2. Results

### 2.1. Specificity of Primers

**Figure 1:**
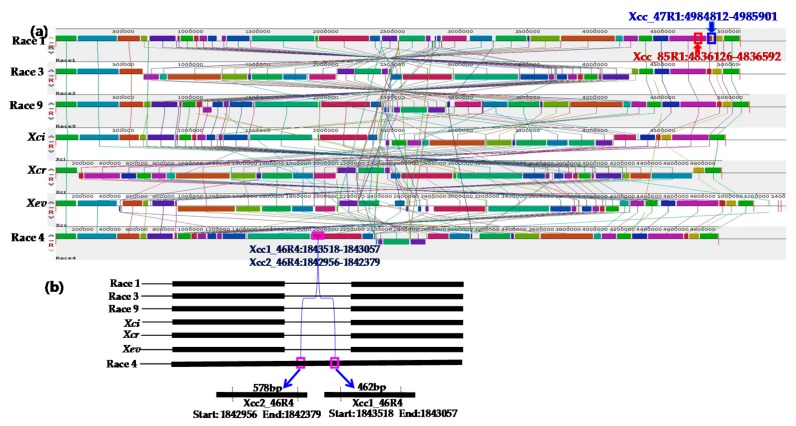
Alignment of whole genome sequences of four *Xanthomonas campestris* pv. *campestris* races, two other *X. campestris* pathovars (*incanae* and *raphani*) and another *Xanthomonas* species (*X. euvesicatoria*). (**a**) A multiple alignment of whole-genome of four races (races 1, 3, 4 and 9) of *Xcc* and three *X. campestris* pathovars/species (*Xci*, *Xcr and Xev*) consists of several rearranged pieces larger than 1 Kb. Each genome is laid out horizontally with homologous segments (LCBs) outlined as colored rectangles. Regions inverted relative to race 1 (B100) are set below those that match in the forward orientation. Lines collate aligned segments between genomes. Average sequence similarities within an LCB, measured in sliding windows, are proportional to the heights of interior colored bars. Large sections of white within blocks and gaps between blocks indicate lineage specific sequence; (**b**) Comparative homology among four races and three *X. campestris* pathovars/species using Mauve tool, version 2.4.0 and Geneious software (Free trial version).

**Figure 2:**
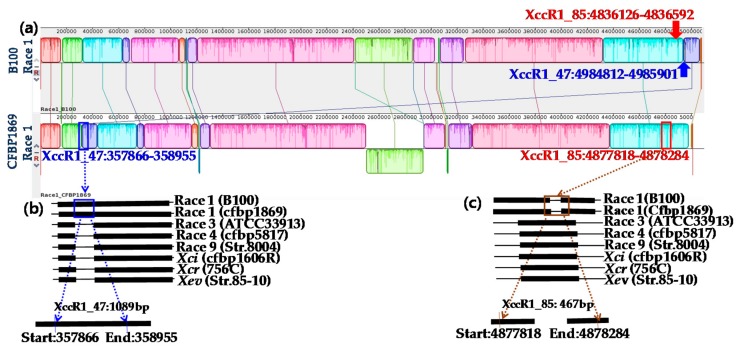
(**a**) A Mauve tool (version 2.4.0)-based visualization of two strains of *Xanthomonas campestris* pv. *campestris* race 1 (B100 and CFBP1869). Comparative homology of published genome sequences of *Xcc* and other *Xanthomonas campestris* pathovars developed using Geneious 8.0 (free trial) software for two race 1 specific designed primers (**b**) Xcc_47R1 and (**c**) Xcc_85R1.

**Figure 3:**
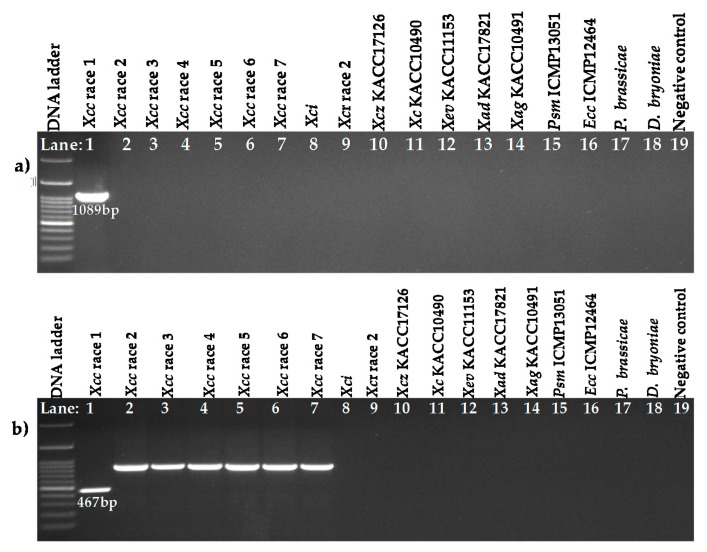
Agarose gel electrophoresis of PCR products from genomic DNA of *X. campestris* pv. *campestris* (*Xcc*) races, *X. campestris* pathovars (*incanae* and *raphani*) and other bacteria, protist and fungus. Reactions were performed using the primer pairs (**a**) Xcc_47R1_F, Xcc_47R1_R and (**b**) Xcc_85R1_F, Xcc_85R1_R. DNA ladder-100 bp; Lanes 1–7: *Xcc* race 1 to race 7; Lane 8: *Xc*i (WHRI-6377); Lane 9: *Xcr* (WHRI-8305); Lane 10: *Xcz* (KACC17126); Lane 11: *Xc* (KACC10490); Lane 12: *Xev* (KACC11153); Lane 13: *Xad* (KACC17821); Lane 14: *Xag* (KACC10491); Lane 15: *Pseudomonas syringae* pv. *maculicola* (ICMP13051); Lane 16: *Erwinia carotovora* subsp. *carotovora* (ICMP12464); Lane 17: *Plasmodiophora brassicae*, Lane 18: *Didymella bryoniae* and Lane 19: Negative control (DDW); gDNA concentration of all samples was 60 ng µL^−1^.

Each of the similar blocks representing sequence homology were shown by a single colour (Figure 1 and Figure 2). Any dissimilarity between sequences was targeted to develop markers (Figure 1 and Figure 2). The primer pair Xcc_47R1 (forward and reverse), designed from *Xcc* race 1 sequences, amplified a fragment of 1089 bp from only one sample tested (*Xcc* race 1 strain, Lane 1, Figure 3a). In contrast, another primer combination, Xcc_85R1, amplified all DNA samples, from all races of *Xcc* (Figure 3b). However, the DNA sample from race 1 produced an expected amplicon size of 467 bp, whereas those of other six races was close to 900 bp (expected product size is 872 bp as per calculations) and were identical to each other (Figure 3b). In both cases, none of the other tested DNA samples of *X. campestris* pathovars, other bacteria species, fungal species and protist produced any visible amplicons (Figure 3a,b).

**Figure 4:**
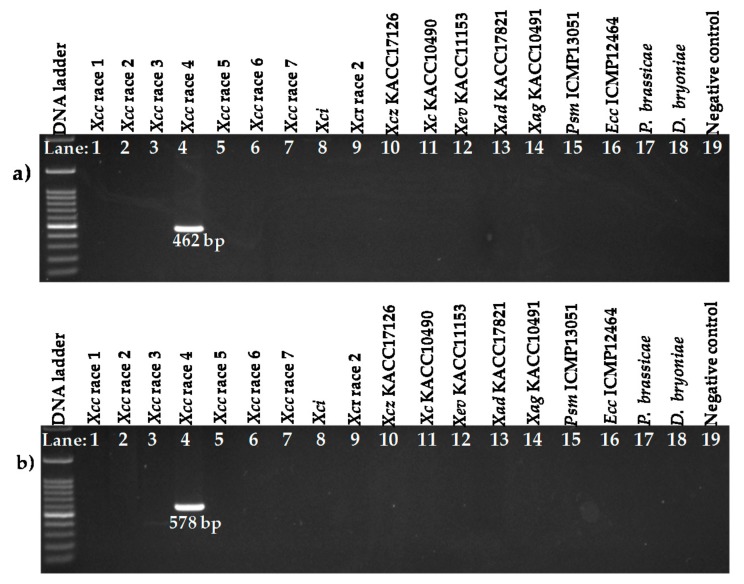
Agarose gel electrophoresis of Sequence Characterized Amplified Regions (SCAR) PCR products from genomic DNA of *X. campestris* pv. *campestris* races and *X. campestris* pathovars and other bacteria and protist. Reactions were performed using the primer pairs (**a**) Xcc1_46R4_F, Xcc1_46R4_R and (**b**) Xcc2_46R4_F, Xcc2_46R4_R. DNA ladder-100 bp; Lanes 1–7: *Xcc* race 1 to race 7; Lane 8: WHRI-6377 (*Xci*); Lane 9: WHRI-8305 (*Xcr*); Lane 10: *Xcz* (KACC17126); Lane 11: *Xc* (KACC10490); Lane 12: *Xev* (KACC11153); Lane 13: *Xad* (KACC17821); Lane 14: *Xag* (KACC10491); Lane 15: *Pseudomonas syringae* pv. *maculicola* (ICMP13051); Lane 16: *Erwinia carotovora* subsp. *carotovora* (ICMP12464); Lane 17: *Plasmodiophora brassicae*, Lane 18: *Didymella bryoniae* and Lane 19: Negative control (DDW). g DNA concentration of all samples was 60 ng µL^−1^.

Two primer pairs designed from an *Xcc* race 4 sequence, Xcc1_46R4 and Xcc2_46R4, also amplified only from the DNA sample of race 4 (*Xcc* race 4 strain, Lane 4, Figure 4a,b). None of the other samples were amplified by these two sets of primers.

### 2.2. Validation of Markers through Bio-PCR in Inoculated Cabbage Leaves

**Figure 5:**
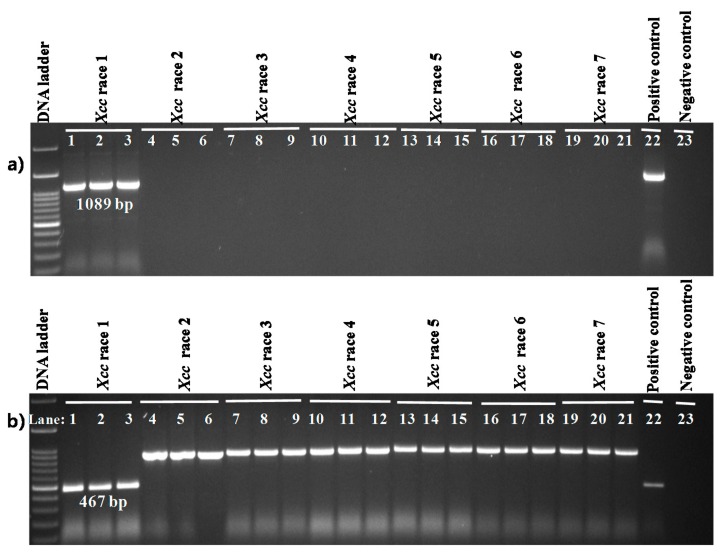
Bio-PCR detection of *Xcc* race 1 in cabbage leaves using two race 1 specific markers (**a**) Xcc_47R1 and (**b**) Xcc_85R1; Lanes 1–21: Three inoculated leaf samples against each of seven *Xcc* races (races 1–7); Lane 22: Control HRIW-3811-genomic DNA (race 1) and Lane 23: Negative control (DDW).

The primer pair Xcc_47R1 designed for race 1 showed positive bio-PCR reaction only for the three samples that were infected with race 1 out of 21 inoculated leaf samples (3 samples from plants inoculated with each race) (Figure 5a). The expected amplicons of 1089 bp specific for the *Xcc* race 1 were observed (Figure 5a) in three race 1 inoculated cabbage leaf samples. Other 18 samples that were inoculated with six other *Xcc* races did not produce any observable amplicons. The positive control DNA sample (in Lane 22) derived from *Xcc* race 1 produced identical amplicons as the amplicons obtained from *Xcc* race 1 inoculated cabbage leaf samples (Figure 5a,b). Another primer combination, Xcc_85R1, produced the expected 467 bp amplicons for three bacterial samples from plants inoculated with race 1; bacterial samples from the plants inoculated with the other six races resulted in identical amplicons of expected 872 bp in size (Figure 5b).

**Figure 6:**
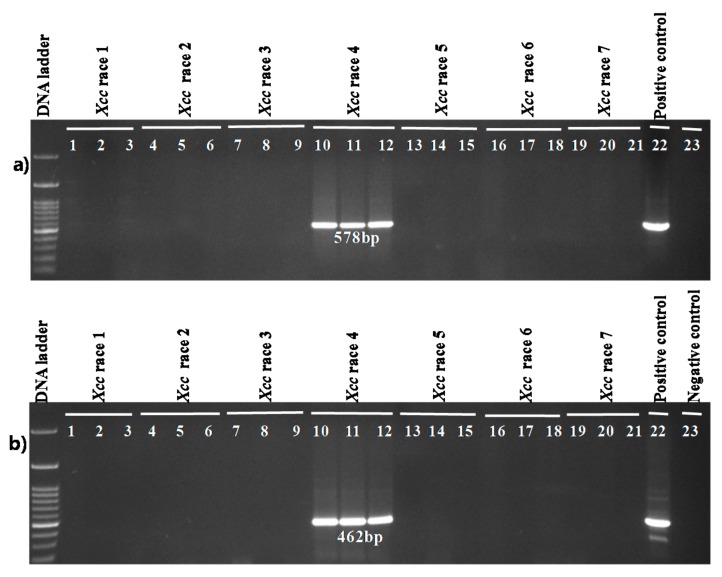
Bio-PCR detection of *Xcc* race 4 in cabbage leaves shown using two race 4 specific markers (**a**) Xcc1_46R4 and (**b**) Xcc2_46R4; bp; Lanes 1–21: Three inoculated leaf samples against each of seven *Xcc* races (race 1–7); Lane 22: genomic DNA (race 4, HRIW-1279A) and Lane 23: Negative control (DDW).

Similarly, in a separate Bio-PCR with primers designed for race 4, only three samples of plants inoculated with race 4 were amplified and none of the other 18 samples that were infected with six other different races yielded any bands (Figure 6a,b). As expected, the control DNA sample was amplified that confirmed the presence of a race 4 sample.

### 2.3. Race Determination

**Figure 7:**
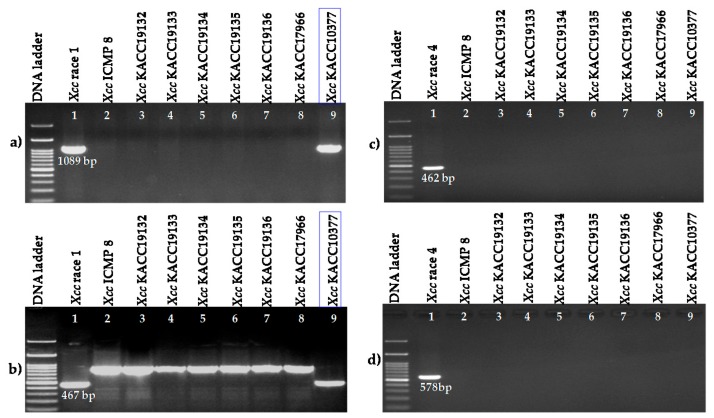
PCR amplification of race-unknown strains of *X. campestris* pv. *campestris* for race determination with two race 1-specifc markers: (**a**) Xcc_47R1 and (**b**) Xcc_85R1; Lane1: HRIW-3811 (*Xcc* race 1 control); and with two race 4-specific markers (**c**) Xcc1_46R4 and (**d**) Xcc2_46R4 Lane1: HRIW-1279A (*Xcc* race 4 control).

Two race 1 specific markers (Xcc_47R1 and Xcc_85R1) amplified Korean KACC10377 strain with specific bands similar to the control DNA samples of HRIW-3811 strain (Figure 7a,b). Two primer pairs designed for race 4 (Xcc_47R1 and Xcc_85R1) did not amplify from any of the tested unknown strains (Figure 7c,d).

## 3. Discussion

### 3.1. PCR-Based Markers Specifically Detected Xcc Races 1 and 4

The purpose of this study was to develop PCR-based SCAR markers for the identification of *Xcc* races 1 and 4 strains. Molecular methods of race identification have been used for other pathogens. For example, K3a race was identified for *Xanthomonas oryzae* pv. *oryzae* (*Xoo*) bacterial blight pathogen of rice by using an AFLP-derived marker [33]. SCAR markers were found highly reliable to detect CYR32 and CYR33 races of wheat stripe rust, caused by *Puccinia striiformis* f. sp. *tritici*, in China [34]. A modified SCAR marker, inter-retrotransposon Sequence-Characterized Amplified Regions (IR-SCAR), was used to detect race 1 of *Fusarium oxysporum* f. sp. *lactucae* in Lettuce [35]. To date, no race specific markers have been reported for detecting any of the *Xcc* races. Therefore, the development of PCR markers which can be comparatively cheap, reliable and effective for the rapid detection of bacterial pathogens, was the main aim of this study.

### 3.2. Whole Genome Sequences of Xcc Enabled Developing Race-Specific Novel Markers

Whole genome sequences of four races of *Xcc* (1, 3, 4, race 9), two other pathovars of *X. campestris* (*Xci, Xcr*) and another species of *Xanthomonas (Xev*) were used for in silico analysis (www.ncbi.nlm.nih.gov) and enabled us to realign, compare and find variable regions present among them. These genome sequences are a valuable resource for comparative analysis among the different races of *Xcc* (Table 1 [36,37,38,39,40,41,42], Figure 1 and Figure 2). Sequences of *Xcc* races were generally highly conserved. When the homology of different races and pathovars was compared by using a comprehensive suite of molecular biology and NGS analysis tools, our target races, (*Xcc* races 1 and 4) showed some unique sequences at different locations compared to all other races and pathovars. The variable sequences facilitated developing *Xcc* races 1 and 4 markers.

One set of SCAR markers (Xcc_47R1) and one set of InDel marker (Xcc_85R1) have been developed for specific detection of race 1. Similarly, two other SCAR markers Xcc1_46R4 and Xcc2_46R4 were able to distinguish *Xcc* race 4 from all other races of *Xcc* and *X. campestris* pathovars. Thus, three SCAR markers and an InDel marker appeared to be unique for the detection of the *Xcc* race 1 and race 4 isolates. PCR amplification exhibited positive amplicons only when DNA of the targeted *Xcc* race 1 and 4 were used and was negative for other *Xcc* races, *X. campestris* pathovars and other species. The sequences used for primer development were not from the strains used in the tests presented here. Thus, the developed SCAR and InDel markers allowed the identification of a strain of race 1 and a strain of race 4 and they may generally act as a diagnostic marker for *Xcc* races 1 and 4. These markers will have to be extensively tested with a larger number of strains of each *Xcc* race, to assess specificity and determine if they detect all *Xcc* race 1 and *Xcc* race 4 strains or just a subset of strains of these races.

There is no information on *Xcc* races in Korea, but *Xcc* is prevalent in this country. We believe that these PCR-based assays with race-specific SCAR and InDel markers have potential for identification of *Xcc* races 1 and 4 of this pathogen from black rot infected plant samples that might also be contaminated with several other bacteria, fungi and microbes.

### 3.3. Direct and Rapid Detection Tool for Xcc Race 4 Developed

A Bio-PCR method was previously exploited for the amplification of a member of the *rhs* family gene for detecting *X. oryzae* pv. *oryzae* (*Xoo*) at the pathovar and species levels [43]. In our study, we tested this method in cabbage leaves inoculated with seven different races. This approach was found very effective and accurate. These results suggested that the PCR-based technique can be used directly to detect and identify *Xcc* races 1 and 4 pathogens in infected cabbage leaf samples without isolating the bacteria from the infected leaves. Thus, this method could potentially be used in disease forecasting to assist cabbage growers.

### 3.4. Potential Applications of Race 4 Specific Markers

The race-specific markers can identify races through Bio-PCR within a few hours whereas use of differential cultivars for race determination requires several months [20]. Thus, in a given crop season, a whole crop might fail even before the causal *Xcc* race is identified by using a conventional race-determination method that uses differential cultivars. The bio-PCR assay based on SCAR and InDel markers could be useful and invaluable for the identification of race 1 and 4 strains and can assist in programmes that aim at developing resistant cultivars for the effective control of black rot disease.

Two race 1-specific markers amplified from the Korean strain KACC10377 indicating that this strain might belong to *Xcc* race 1. This is the first identified race in Korea. In Asia, race 1, race 4 and race 6 are dominant in Nepal [5] and India [32]. Thus, the *Xcc* strain identified in this study could be part of an important *Xcc* race in Korea.

The markers developed will need to be tested with a large collection of *Xcc* isolates to assess their specificity and the method should also be tested with plants at different stages of growth and infection. It will be particularly important to determine if the strains can be detected early after infection and before symptom development.

## 4. Materials and Methods

### 4.1. Bacterial Strains and Culture Conditions

Representative strains of *Xcc* races (races 1 to 7) and *X. campestris* pathovars HRIW-6377 (*Xci*), HRIW-8305 (*Xcr*) were obtained from the culture collection of the School of Life Sciences, Wellesbourne Campus, University of Warwick, UK (HRIW). Three *Xcc* isolates ICMP8, ICMP13051 and ICMP12464 were obtained from the Landcare Research, Auckland, New Zealand (Table 2 [3,44]). Other eight *Xcc* (KACC19132, KACC19133, KACC19134, KACC19135, KACC19136, KACC10377 and KACC17966) and *X. campestris* isolates (KACC11153, KACC11154, KACC17821, KACC17126, KACC10491, KACC10490) were obtained from the Korean Agriculture Culture Collection (KACC) (Table 2 [3,44]). All bacterial isolates were cultured on King’s medium B [45] and incubated at 30 °C for 48 h.

### 4.2. Isolation of Total DNA

Genomic DNA of all bacteria isolates was extracted using a DNeasy Plant Mini Kit (QIAGEN, Hilden, Germany) following the Manufacturer’s instructions. The concentration and purity of the extracted DNA was then measured using a Nanodrop ND-1000 spectrophotometer (NanoDrop, Wilmington, DE, USA).

### 4.3. Sequence Retrieval and Alignment

In silico analysis was conducted for designing primers for specific detection of strains of *Xcc* races 1 and 4. For comparative genome analyses, whole genome sequences of *Xcc* strains of B100 (race 1), CFBP1869 (race 1), ATCC33913 (race 3), CFBP5817 (race 4) and 8004 (race 9) and other three *Xanthomonas* pathovars/species (*Xanthomonas campestris* pv. *incanae*‒*Xci* (CFBP1606R), *X. campestris* pv. *raphani‒Xcr* (756C) and *X. euvesicatoria‒Xev* (strain 85-10) were downloaded from NCBI [46]. The genome size of *Xcc* races and *X. campestris* pathovars ranged between 4.91 Mbp and 5.17 Mbp (Table 2) and their GC content ranged between 64.2 and 65.3 indicating that there might be large nucleotide sequence variation among them. For comparative purpose, the strain B100 (race 1) was considered as a reference genome for designing primers. Mauve (version 2.4.0) and Geneious (Free trial version) were used to align the genomes and to identify the homology between sequence blocks among the races of *Xcc* (shown by differential colors); this facilitated the identification of differential regions among these isolates that were useful to develop primers for specific races.

### 4.4. Primer Design and PCR Conditions

The primers were designed from whole genome sequences of bacteria using Primer3 software [47] and checked for specificity in silico using ‘In Silico simulation of molecular biology experiments software’ (www.insilico.ehu.es). Highly conserved regions were excluded when primers were designed. Only variable regions were selected as they may provide race-specific signature sequences required for the identifying *Xcc* races 1 and 4. Four sets of potential race-specific primers were designed, two sets for race 1 and two sets for race 4. Primers Xcc_47R1 and Xcc_85R1 were designed to detect race 1 and primers Xcc1_46R4 and Xcc2_46R4 were designed to detect race 4 (Table 3). The race 1 primers were: Xcc_47R1 between 498412-4985901 and Xcc_85R1 between 4836126–4836592; these two primers were expected to amplify 1089 and 467 base pairs sequences, respectively. The race 4 specific primers were: Xcc1_46R4 designed between 1843518 and 1843057 and Xcc1_46R4 primer designed between 1842956 to 1842379; these two primers were expected to amplify 462 and 578 base pairs, respectively (Table 3).

Emerald PCR master mix (Takara, Shiga, Japan) was used in Polymerase Chain Reaction (PCR) for amplification of the target regions with respective markers. A 20.0 μL of PCR reaction mixture containing forward and reverse primers (1.0 μL each), Emerald PCR master mix (9.0 μL), ultra-pure water (8 μL) and 1.0 μL DNA was used for PCR amplification. PCR was performed using the following conditions in a thermo cycler (Takara, Shiga, Japan): denaturation at 95 °C for 5 min followed by 20 cycles of amplification at 95 °C for 30 s, annealing at 65 °C for 40 s and 72 °C for 45 s, and terminated by a final elongation at 72 °C for 5 min, but in case of race 4-specific amplification annealing was carried out at 66 °C for 40 s. Electrophoresis was done using 1.5% agarose gel at 100 V for 30 min, visualized on ENDURO^TM^ *GDS Gel Documentation system* under UV light (302 nm).

### 4.5. Testing the Specificity of Primers

At first, the specificity of the designed primers was checked by using BLAST tool [48]. Wet lab validation was carried out by performing PCR with genomic DNA (1 μL of a suspension approximately at 60 ng μL^−1^) of *Xcc* races (races 1 to 7) and eight *Xcc* strains (of unknown race), two other pathovars of *X. campestris* (*Xci* and *Xcr*), five strains of other *Xanthomonas* species (two strains of *X. euvesicatoria*, *X. campestris* pv. *zinniae*, *X. axonopodis* pv. *dieffenbachiae and X. axonopodis* pv. *glycines*) and strains of other test-bacteria species e.g., *Pseudomonas syringae* pv. *maculicola, Erwinia carotovora* subsp. *carotovora,* one fungal species, *Didymella bryoniae*, and a protist *Plasmodiophora brassicae*. The precision and specificity of the developed primers Xcc_47R1 and Xcc_85R1 for race 1, and Xcc1_46R4 and Xcc2_46R4 for race 4 detection (Table 2 [3,44]) were assessed. Negative controls were performed with the DNA template replaced with a 1.0 μL DDW.

### 4.6. Detection of the Race 1 and 4-Specific Pathogen by PCR in Artificially Infected Cabbage Leaves

The sensitivity of the four specific markers (i.e., their efficiency) in direct PCR based assay was evaluated using artificially infected leaves. For evaluation of these primers, 35-days-old cabbage inbred line (BN3122, Asia Seed Co. Ltd., Seoul, Korea) plants, susceptible against black rot disease, were inoculated with seven races of *Xcc* (Table 1) in a glasshouse with artificially controlled temperature (26 ± 2 °C) and relative humidity (70–80%). Three different individual plants were inoculated with each race. The inoculated plants that had visible and conspicuous black rot symptoms at 14 days after inoculation (DAI) were sampled. V-shaped lesions with blackened veins of about 2 cm were cut into small pieces and each cut piece of sample was soaked in 200 μL ultra-pure water. After 40 min, 10 μL water containing bacterial cells was taken for PCR reactions. The bio-PCR was performed following the method as described in ‘PCR amplification’ with an exception in number of amplification cycles (30 cycles).

### 4.7. Race Identification

Genomic DNA samples of eight *Xcc* strains of unknown race (ICMP8, KACC19132, KACC19133, KACC19134, KACC19135, KACC19136, KACC17966 and KACC10377) were used for race identification. PCR amplification was done using developed molecular markers for both race 1 and 4 for specific detection of races of *Xcc*. Two strains of two known races, strain HRIW-3811 of race 1 and strain HRIW-1279A of race 4 were used as controls.

## 5. Conclusions

This study exploited the variation within the whole genome sequences of *Xcc* races and pathovars for developing *Xcc* races 1 and 4 specific markers. Markers developed based on in silico analysis were further tested for their specificity through BLAST search. Four markers were able to distinguish *Xcc* races 1 and 4 from all other *Xcc* races and pathovars. The developed markers have potential to identify *Xcc* races 1 and 4 from any infected cabbage leaf samples rapidly and effectively without deploying a DNA isolation procedure. This is the first report that develops *Xcc* race 1 and 4-specific markers for the direct and rapid detection of black rot disease. These markers could be utilized worldwide for early detection of black rot disease in infected fields and for quick identification of *Xcc* races.

## Figures and Tables

**Table 1:** Published whole genome sequences of *Xcc* races and *Xc* pathovars with accession number, genome size and GC content.

Strains	Accession	Races	Genome Size (bp)	G + C Content (%)	Reference
ATCC 33913 (UK) *Xanthomonas campestris* pv. *campestris*	AE008922	3	5,076,188	65.1	[36]
Strain 8004 *Xanthomonas campestris* pv. *campestris*	NC_007086	9	5,148,708	65.0	[37]
B100 (UK) *Xanthomonas campestris* pv. *campestris*	AM920689	1	5,079,002	65.0	[38]
CFBP1869 (France) *Xanthomonas campestris* pv. *campestris*	NZ_CM002545	1	5,008,832	65.0	[39]
CFBP 5817 (France) *Xanthomonas campestris* pv. *campestris*	NZ_CM002673	4	4,918,955	65.2	[39]
CFBP1606R *Xanthomonas campestris* pv. *incanae*	NZ_CM002635	-	4,967,288	65.0	[40]
756C *Xanthomonas campestris* pv. *raphani*	NC_017271	-	4,941,214	65.3	[41]
Strain 85–10 *Xanthomonas euvesicatoria*	NC_007508.1	-	5,178,466	64.7	[42]

**Table 2:** Plant pathogenic bacterial races of *Xanthomonas campestris* pv. *campestris*, *X. campestris* pathovars/species and other strains used in this study with origin, host and year of collection.

SL.	Bacterial Strains *	Races	Host	Country	Collection Year	Reference
1	*X. campestris* pv. *campestris* (HRIW-3811)	1	*B. oleracea*	US	2017	[3]
2	*X. campestris* pv. *campestris* (HRIW-3849A)	2	*B. oleracea* var. *botrytis*	US	2017	[3]
3	*X. campestris* pv. *campestris* (HRIW-5212)	3	*B. oleracea* var. *gemmifera*	UK	2017	[3]
4	*X. campestris* pv. *campestris* (HRIW-1279A)	4	*B. oleracea* var. *capitata*	UK	2017	[3]
5	*X. campestris* pv. *campestris* (HRIW-3880)	5	*B. oleracea* var. *capitata*	Australia	2017	[3]
6	*X. campestris* pv. *campestris* (HRIW-6181)	6	*B. rapa*	Portugal	2017	[3]
7	*X. campestris* pv. *campestris* (HRIW-8450A)	7	*B. oleracea* var. *capitata*	UK	2017	[3]
8	*X. campestris* pv. *campestris* (ICMP8)	-	*Brassica oleracea* var. *capitata*	New Zealand	2016	This work
9	*X. campestris* pv. *campestris* (KACC19132)	-	*B. rapa* (Pyeongchang)	South Korea	2017	This work
10	*X. campestris* pv. *campestris* (KACC19133)	-	*B. rapa* (Gangneung)	South Korea	2017	This work
11	*X. campestris* pv. *campestris* (KACC19134)	-	-	South Korea	2017	This work
12	*X. campestris* pv. *campestris* (KACC19135)	-	-	South Korea	2017	This work
13	*X. campestris* pv. *campestris* (KACC19136)	-	-	South Korea	2017	This work
14	*X. campestris* pv. *campestris* (KACC10377)	-	*Brassica oleracea* var. *capitata*	South Korea	2017	This work
15	*X. campestris* pv. *campestris* (KACC17966)	-	-	South Korea	2017	This work
16	*X. campestris* pv. *incane* (WHRI-6377)	-	*Matthiola incana*	UK	2017	[3]
17	*X. campestris* pv. *raphanin* (WHRI-8305)	2	*B. rapa* var. *perviridis*	UK	2017	[3]
18	*X. campestris* (KACC10490)	-	-	South Korea	2017	This work
19	*Pseudomonas syringae* pv. *maculicola* (ICMP13051)	-	*Brassica oleracea* var. *capitata*	New Zealand	2016	This work
20	*Erwinia carotovora* subsp. *carotovora* (ICMP12464)	-	*Brassica oleracea* var. *capitata*	New Zealand	2016	This work
21	*Plasmodiophora brassicae* (Pathotype1)-Gangneung-1	-	*B. rapa*	South Korea	2016	[44]
22	*X. euvesicatoria* (KACC11153)	-	-	South Korea	2017	This work
23	*X. axonopodis* pv. *dieffenbachiae* (KACC17821)	-	*Anthurium andraeanum* (Yongin)	South Korea	2017	This work
24	*X. campestris* pv. *zinniae* (KACC17126)	-	*Zinnia elegans* (Suwon)	South Korea	2017	This work
25	*X. axonopodis* pv. *glycines* (KACC10491)	-	*Glycine max*	South Korea	2017	This work
26	*Didymella bryoniae* (NIHHS1326)	-	*Cucumis melon*	South Korea	2016	This work

* ICMP: International Collection of Microorganisms from Plants (ICMP), Landcare Center, Auckland, New Zealand; KACC: Korean Agricultural Culture Collection, Korea; NIHHS: National Institute of Horticultural and Herbal Science, Korea; HRIW: collection from the School of Life Sciences, Wellesbourne Campus, The University of Warwick, UK.

**Table 3:** Primer sets used for *Xcc* race 1 and race 4-specific PCR amplification.

Primer Name	Sequences (5’…3’)	Genomic Position	Gene Name	Description	Base Pair (bp)	Annealing Temperature
Xcc_47R1_F	CCTCCTGAGTCATGGCAATGGC	498412-4985901	xcc-b100_4389	Peptidoglycan binding Protein	1089	65 °C for 40 s
Xcc_47R1_R	TAGCAGGGGAGTGCTGCTTGC					
Xcc_85R1_F	GCGGCTCGGCTTCACGGTCAGC	4836126-4836592	xcc-b100_4275	Membrane protein with arac family transcriptional regulator and peptidase domain	467	
Xcc_85R1_R	GCCCAGGATGCAGCGCAGCGT					
Xcc1_46R4_F	GGCATGGGGAATGATCGTTGAC	1843518-1843057	Intergenic	-	462	66 °C for 40 s
Xcc1_46R4_R	ATGCGGGCGATGGGATGGCCA					
Xcc2_46R4_F	GCGTAGCGAAAACTGGTAGTTC	1842956-1842379	Intergenic	-	578	
Xcc2_46R4_R	GCACAGGCGCACCAGCATATGGC					

## Supplementary Materials

The following are available online at www.mdpi.com/1422-0067/18/12/2523/s1.

## Abbreviations

*Xcc*	*Xanthomonas campestris* pv. *campestris*
*Xci*	*Xanthomonas campestris* pv. *incane*
*Xcr*	*Xanthomonas campestris* pv. *raphani*
PCR	Polymerase chain reaction
SCAR	Sequence-characterized amplified region
InDel	Insertions or deletions
