# Supplementary material for: Whole-Genome Re-Alignment Facilitates Development of Specific Molecular Markers for Races 1 and 4 of Xanthomonas campestris pv. campestris, the Cause of Black Rot Disease in Brassica oleracea
Source: Int J Mol Sci. 2017 Nov 24;18(12):2523. doi: 10.3390/ijms18122523 (PMC5751126; doi:10.3390/ijms18122523)
Supplement: Supplementary file 1 [file ijms-18-02523-s001.pdf]

Supplementary Materials: Whole-genome re-alignment facilitate development of specific molecular markers for races 1 and 4 of *Xanthomonas campestris* pv. *campestris*, the cause of Black rot disease in *Brassica oleracea*

Mehede Hassan Rubel, Arif Hasan Khan Robin, Sathishkumar Natarajan, Joana G. Vicente, Hoy-Taek Kim, Jong-In Park, Ill-Sup Nou

Table S1. Resistance accessions developed in *Brassica olerecea.*

Sl	Cultivars	Resistant against races	*Brassica* species	Country	Reference
1	PI436606	2	*Brassica oleracea*	USA	[14]
2	SI, Badger Inbred 16, Kinkei DHOI , Reiho DHOl , Fujiwase I; Fujiwase DHOI, 02, 05; Harukei DHOI	1 and 2			
3	DH M9606; Aichi dai Bansei DHOl, 02	race 2	*Brassica oleracea*	Russia	[15]
4	Reiho DHOI , 03; Fujiwase DH03, 04; Matsunami DH22	2 and 4			
5	Matsunami DH77; DH M9602, 9603	4			
6	Miracle F1	2, 3, 5, 8 and 9	*Brassica oleracea*	UK	[3,10]
6	Three cultivars	10 and 11	*Brassica oleracea*	Portugal	[11]

Table S2. Differential cultivars used for detection of *Xcc* races (adapted from Vicente and Holub, 2013).

Sl	Differential Cultivars	Resistant against races	Species	Reference
1	Wirosa F1	Susceptible	*Brassica oleracea*	[3,5,9,10,11,32]
2	Just Right Hybrid Turnip	4 and 9	*Brassica rapa*	
3	Cobra (COB60)	4 and 9	*B. napus*	
4	Seven Top Turnip	2, 8 and 9, but variable resistance to races 4, 5	*Brassica rapa*	
5	Florida Broad Leaf Mustard (FBLM2)	1, 3, 4, 7, 8 and 9	*Brassica juncea*	
6	PI 199947 (PIC1)	1, 3, 4, 8 and 9	*B. carinata*	
7	Miracle F1	2, 3, 5,8 and 9	*B. oleracea*	
